# Commentary: The Attraction Effect in Decision Making: Superior Performance by Older Adults

**DOI:** 10.3389/fpsyg.2018.02321

**Published:** 2018-11-22

**Authors:** Maciej Koscielniak, Klara Rydzewska, Grzegorz Sedek

**Affiliations:** ^1^Interdisciplinary Center for Applied Cognitive Studies, Warsaw, Poland; ^2^Faculty in Poznan, SWPS University of Social Sciences and Humanities, Poznań, Poland; ^3^Department of Psychology, SWPS University of Social Sciences and Humanities, Warsaw, Poland

**Keywords:** aging, attraction effect, decoy effect, asymmetric dominance, decision making

Older adults are stereotypically perceived as more susceptible to various scams and manipulations (Cuddy et al., 2005). However, this hardly finds confirmation in scientific literature, where older citizens are often portrayed as very resistant to social and consumer influence. In some areas they even outperform their younger counterparts and preserve high effectiveness in decision-making until late adulthood (Yoon and Lee, 2004; Ross et al., 2014; Berg, 2015).

One of the examples of common consumer manipulation is the *attraction effect* (also known as the *decoy effect* or *asymmetric dominance effect*; Huber et al., 1982). It describes the case of preference reversals induced by the introduction of a decoy (inferior) option to a set of original alternatives. As a result, the dominating alternative (similar to the decoy but noticeably better) attracts attention and its popularity increases. This phenomenon has often been conceived as an example of irrationality in human choices and a clear violation of utility theory (von Neumann and Morgenstern, 1944) (according to which preferences should be independent of irrelevant options; Luce, 1959).

The attraction effect is not only associated with people's economic choices, but also with a wide range of social decisions and judgements (Trueblood et al., 2013). The effect can be observed as soon as children start to appreciate the relative values of alternatives (at around age 5; Zhen and Yu, 2016); but little is known about its effectiveness in late adulthood. There are only two papers investigating this topic (Tentori et al., 2001; Kim and Hasher, 2005)—both suggesting that older adults are less prone to be victims of manipulation based on the presence of dominated options.

The value of those articles is unquestionable, as they give a solid rationale to confront popular and harmful stereotypes about aging. The aim of this commentary is not to undermine those results, but to suggest an alternative explanation for the observed lifetime changes in susceptibility to the attraction effect. Our elucidation may also be helpful in understanding why the effect of age on the attraction effect is strong in some studies, but insignificant in others.

Tentori et al. (2001) explain the results of their experiment in terms of the prudency and rationality of older adults, who deliberately exclude unfavorable options from their choice (in contrast to younger adults). Kim and Hasher (2005) expands this by focusing on the role of life-experience, reliance on different processing styles, and Damasio's somatic markers. All of these explanations may actually play an important role in the susceptibility to the attraction effect in late adulthood. However, we believe that they should be extended by lifetime changes in fluid competences and in epistemic motivation, which are crucial for most types of decision making in elderly adults.

It is empirically confirmed that adults experience monotonic decrease in basic cognitive abilities (incl. mental speed, working memory capacity, etc.) throughout their life (Salthouse, 2012; Sedek et al., 2013). The engagement in demanding mental processes is related to increasing cognitive costs (Hess, 2014), so older adults become very selective in choosing tasks in which they want to invest their limited resources (Baltes and Baltes, 1990). We strongly believe that this mechanism is important for analyzing the susceptibility to the attraction effect in older adults, who may avoid engaging in hypothetical scenarios used in laboratory settings.

We predict that older adults may not be able to notice the character of the dominating relation, which makes them resistant to the attraction effect. This hypothesis is fully consistent with the recent theoretical model of Crosetto and Gaudeul (2014), who claims that there are two necessary steps for this effect to occur: to perceive the comparable similarity between the dominant option and the decoy; and to notice the dominant relation between them (leading to the exclusion of decoy). We suggest that due to the mentioned cognitive and motivational limitations, older adults may not be able to satisfy at least one of these conditions when the attributes are complex and mentally demanding.

In the Supplementary Materials we analyze the relationship between age and the number of decoys chosen in the commented article (as an indicator of the inability to notice the dominant relation in the choice set). The Chi-Squared Tests of Independence show that older adults choose the decoys more frequently (in comparison to dominating option choices) only in the grocery task (where only young adults showed the attraction effect), while no such relationship is confirmed for extra-credit task (no attraction effect in both age groups).

Our explanation portrays the paradoxically positive impact of cognitive deficits on decision-making quality in late adulthood. Older adults may be protected from complex context manipulations due to cognitive declines and their unwillingness to invest cognitive resources in effortful decision processes. This is a promising starting point for further studies on lifetime changes in susceptibility to the attraction effect and other context effects. We expect that those changes will be especially distinct for decisions requiring certain amount of deliberation, but will not be significant for tasks described by simple, perceptual attributes (or demanding experience-based knowledge). In the domains of automatic and intuitive decision-making there are no lifetime competence declines (Mikels et al., 2010), so the decoy alternative should be perceived similarly by younger and older adults.

To conclude, the proposed explanation of lifetime changes in susceptibility to the attraction effect refers to cognitive and motivational changes in late adulthood (Hess, 2014) and to dual system theories (Stanovich and West, 2000; Kahneman, 2003). We expect that for decisions based on deliberative stimuli (requiring numeric or quality information processing) older adults will be more resistant to context manipulations compared to younger adults—as described by Tentori et al. (2001) and partially by Kim and Hasher (2005). However, for the choices described by System 1 attributes [intuitive or relying on visual perception, as in the study of Trueblood et al. (2013)] we expect to observe no differences. This hypothesis suggests that stereotypically perceived weaknesses of late adulthood (cognitive declines) can also act as compensatory mechanisms in specific situations, leading to more successful decision-making.

## Author contributions

All authors listed have made a substantial, direct and intellectual contribution to the work, and approved it for publication.

### Conflict of interest statement

The authors declare that the research was conducted in the absence of any commercial or financial relationships that could be construed as a potential conflict of interest.
