# Supplementary material for: Commentary: The Attraction Effect in Decision Making: Superior Performance by Older Adults
Source: Front Psychol. 2018 Nov 22;9:2321. doi: 10.3389/fpsyg.2018.02321 (PMC6262069; doi:10.3389/fpsyg.2018.02321)
Supplement: Supplementary file 1 [file Table_1.docx]

Supplementary Material

*Commentary: “The Attraction Effect in Decision Making: Superior Performance by Older Adults”*

**Maciej Koscielniak^1,2^*, Klara Rydzewska^1,3^ and Grzegorz Sedek^1,3^**

^1^Interdisciplinary Center for Applied Cognitive Studies, Warsaw, Poland

^2^SWPS University of Social Sciences and Humanities, Faculty in Poznan, Poland

^3^SWPS University of Social Sciences and Humanities, Department of Psychology, Warsaw, Poland

*** Correspondence:**

Maciej Koscielniak

SWPS University of Social Sciences and Humanities

61-719 Poznan, Poland

mkoscielniak@swps.edu.pl

**Analysis of the Relationship Between Age and Number of Decoy Choices**

Kim and Hasher (2005) found that younger adults showed the attraction effect only in the grocery shopping problem but not in the extra credit problem, whereas older adults did not show the attraction effect for either problem. Similarly to Kim and Hasher (2005), we chose the method of Chi-Square Tests of Independence to examine the relationship between age (younger vs. older adults) and decision preferences (ratio of decoy choices vs. target choices). The descriptive and Chi-Square statistics are presented in Table 1. A significant result was only obtained in the grocery task, suggesting that older adults actually make more irrational choices compared to younger adults. No such effect was observed in the extra credit task. This evidence confirms our hypothesis about the relationship between cognitive abilities and susceptibility to attraction effect. For older adults it is more difficult to notice the dominant relation between decoy and target, what leads to more frequent choices of dominated options and smaller susceptibility to attraction effect.

Table 1

*Description of the choice preferences in Kim and Hasher (2005) experimental tasks (3-choice condition), separately for younger and older adults*

| Task | Age group | Chose target | Chose decoy | Chi-squared statistic | p | φ |
| --- | --- | --- | --- | --- | --- | --- |
| Grocery | Younger adults | 118 | 9 | 4.63 | 0.031 | 0.153 |
|  | Older  adults | 59 | 12 |  |  |  |
| Extra credit | Younger adults | 239 | 25 | 1.45 | 0.229 | 0.042 |
|  | Older  adults | 116 | 18 |  |  |  |

Note. Chosen target = B option; Chosen decoy = C option, φ = mean square contingency coefficient (a measure of association for age and choice preference).
